# Commentary: The fate of germ cells in cryptorchid testis

**DOI:** 10.3389/fendo.2024.1393040

**Published:** 2024-04-12

**Authors:** Feridun Cahit Tanyel

**Affiliations:** Turkish Academy of Sciences, Ankara, Türkiye

**Keywords:** descent of testis, cryptorchidism, infertility, undescended testis, autonomic balance

## Introduction

I read the article by Thorup et al. with interest (1). The authors aim to elucidate the germ cell pathogenesis in cryptorchid testes within the context of infertility and the development of testicular malignancies.

There is no universally accepted mechanism to explain testis descent and the reason for the inhibition of testicular descent. An overlooked explanation to date seems to provide the most satisfactory mechanism of normal and failed descents that links all the associations related to normal or failed procedures (2–4). Elucidating the pathogenesis in germ cells associated with undescended testis through this perspective may help to broaden our understanding of the subject.

## Descent of a testis

The gubernaculum gives rise to smooth (SM) and striated cremaster muscles around the processus vaginalis (PV). After the development of muscles, the gubernaculum no longer exists. Descent is propulsion. The testis is propelled through the PV via peristaltic force. After descending the testis, the SM should undergo programmed cell death for the obliteration of PV. Initiation of programmed cell death occurs through calcium signaling. The intrinsic pathway is activated via a shift in autonomic balance in favor of the parasympathetic tone. The key point in the process of descent is the regulation of the shift in autonomic balance. Silencing of the central catecholaminergic activity appears to play a role. Alterations in timing, intensity, or duration of shift have pathological consequences. A shift before a descent diminishes the amount of SM. An inadequate amount of SM cannot propel the testis, and results in undescended testis. A shift in autonomic tone persists in boys with undescended testes. The most probable mechanism for persistence is the loss of some catecholaminergic neurons. Differences in signal transduction due to a shift form the basis for associated occurrences. Inadequacy in the intensity or duration of a shift results in the rescue of SM. The persistence of SM inhibits the obliteration of PV and gives rise to inguinal hernia or hydrocele depending on the amount of remaining SM. The defined mechanism links and explains all the occurrences associated with the descent of a testis (2–4).

## Discussion

Boys who have undescended testes are subjected to a decrease in sympathetic tone and an increase in parasympathetic tone (2–4). Persistence of signals directed to induce programmed cell death in SM is the reason for the association of a decrease in fertility and an increase in the risk of malignancy, vaso-epididymal anomalies, retraction and ascent, and disturbances in minipuberty among boys with undescended testis.

An initial wave of apoptosis within the testes has been suggested to be necessary for normal spermatogenesis during adulthood (5). The shift in autonomic tone may also act to initiate the physiologic apoptosis within the testes. The persistence of a shift may lead to apoptosis beyond physiologic requirements in the testes and contribute to a decrease in fertility. Increases in cAMP activate the transcription of specific target genes that contain cAMP response elements. Regulation of gene expression by cAMP plays an important role in controlling the proliferation and differentiation of animal cells (6). The decrease in sympathetic tone, thus less of an increase in cAMP levels in boys with undescended testis, explains the infertility. Continuous stimulation of the protein kinase C (PKC) pathway by phorbol esters results in the development of tumors (6). The dominance of the parasympathetic tone, thus continues the stimulation of the PKC pathway, explaining the increase in the risk of malignancy.

The primitive mesenchymal tissue, so-called the gubernaculum, also supports the muscular layer of vaso-epididymal structures. Signals toward inducing programmed cell death in SM explain both the mechanism of vaso-epididymal anomalies and their association with undescended testis (2–4).

The persistence of shifted signaling increases cytosolic calcium. Activation of phospholipase C generates inositol 1,4,5-trisphosphate. Inositol 1,4,5-trisphosphate releases Ca^2+^ from internal stores. The increase in cytosolic calcium, not the hyperactive cremasteric reflex, contracts the striated cremaster muscle (7, 8). Contracted cremaster muscles may subsequently undergo contracture formation. Since retractile testis shares a similar shift in autonomic tone, it should not be regarded in the spectrum of normal but regarded as abnormal.

Catecholamines also play a role in the control of the pulsatile release of gonadotropin-releasing hormone and in the regulation of hypothalamic–pituitary–gonadal axis (9). The silencing of central catecholaminergic activity links the association between undescended testis and defective minipuberty. A cross-talk seems to exist between the sympathetic tone and the regulation and synthesis of androgens. On the other hand, testosterone release is regulated by an efferent neural pathway from the hypothalamus to the testes under the influence of catecholamines that work independently from the pituitary (10). Since alpha-adrenergic receptor inhibition or chemical sympathectomy reduces testosterone concentration, the sympathetic tone has an important role in testosterone release through this pathway (11, 12).

Anencephaly may help to further clarify the subject. Despite the normal levels of human chorinic gonadotropin (HCG), the absence of Leydig cells is always associated with a severe reduction or absence of the gonocytes in boys with anencephaly (13, 14). Furthermore, abnormalities in the epididymis are frequent in anencephalic boys, but penile abnormalities are not encountered (14). On the other hand, ovaries are normal until 34 weeks of gestation resulting from a sexually dimorphic development (13). Germ cells, Leydig cells, and epididymal anomalies in boys with anencephaly closely resemble those encountered in boys with undescended testis. Similarly, 35% of boys with cryptorchidism responded inadequately to HCG stimulation, while 10% did not respond (15). Undescended testes and testes associated with anencephaly may share similar pathways.

Hypogonadotropic hypogonadism is reported to be the most common cause of cryptorchidism (16). However, testis descent is inhibited through early programmed SM cell death and hypogonadism results from central catecholaminergic changes directed to shift the autonomic tone. Therefore, hypogonadism is not a cause for isolated undescended testis, but a result that shares the decrease in sympathetic tone.

The proposed mechanisms place all the variables correctly. Further evaluations through this window may help to clarify the subject.

## Author contributions

FCT: Writing – original draft, Writing – review & editing.
